# A relationship of sorts: gender and auditory hallucinations in schizophrenia spectrum disorders

**DOI:** 10.1007/s00737-021-01109-4

**Published:** 2021-03-20

**Authors:** Stefanie Suessenbacher-Kessler, Andrea Gmeiner, Tamara Diendorfer, Beate Schrank, Annemarie Unger, Michaela Amering

**Affiliations:** 1 Department of Psychiatry and Psychotherapy, Division for Social Psychiatry, Medical University of Vienna, Währinger Gürtel 18-20, 1090 Vienna, Austria; 2 Department of Psychiatry, Karl Landsteiner University of Health Sciences, Dr.-Karl-Dorrek-Straße 30, 3500 Krems an der Donau, Austria

**Keywords:** Voice hearing, Verbal auditory hallucinations, Schizophrenia spectrum disorders, Gender

## Abstract

Voice hearing has been conceptualized as an interrelational framework, where the interaction between voice and voice hearer is reciprocal and resembles “real-life interpersonal interactions.” Although gender influences social functioning in “real-life situations,” little is known about respective effects of gender in the voice hearing experience. One hundred seventeen participants with a schizophrenia spectrum disorder took part in a semi-structured interview about the phenomenology of their voices and completed standardized self-rating questionnaires on their beliefs about their most dominant male and female voices and the power differentials in their respective voice-voice hearer interactions. Additionally, the voice hearers’ individual masculine/feminine traits were recorded. Men heard significantly more male than female dominant voices, while the gender ratio of dominant voices was balanced in women. Although basic phenomenological characteristics of voices were similar in both genders, women showed greater amounts of distress caused by the voices and reported a persistence of voices for longer time periods. Command hallucinations that encouraged participants to harm others were predominantly male. Regarding voice appraisals, high levels of traits associated with masculinity (=instrumentality/agency) correlated with favorable voice appraisals and balanced power perceptions between voice and voice hearer. These positive effects seem to be more pronounced in women. The gender of both voice and voice hearer shapes the voice hearing experience in manifold ways. Due to possible favorable effects on clinical outcomes, therapeutic concepts that strengthen instrumental/agentic traits could be a feasible target for psychotherapeutic interventions in voice hearing, especially in women.

## Introduction

Verbal auditory hallucinations (VAH) are a core symptom of schizophrenia spectrum disorders and constitute a major source of disease-related distress (Badcock et al. 2011; Kumari et al. 2013) as well as a substantial risk factor for suicidal or otherwise harmful behavior in many affected individuals (DeVylder and Hilimire 2015; Fujita et al. 2015). In recent years, scientists have come to conceptualize voice hearing under an interrelational framework that views the interaction between voice and voice hearer as reciprocal and resembling “real-life interactions” in a number of key characteristics such as social complexity, attachment style, effects of social rank, subordination, and perceptions of power (Benjamin 1989; Birchwood et al. 2000; Hayward 2003; Hayward et al. 2011; Paulik 2012; Robson and Mason 2015; McCarthy-Jones et al. 2015; Upthegrove et al. 2016). Furthermore, seminal research has highlighted that affective responses to voices such as anxiety, distress, and depressive symptoms are strongly influenced by the voice hearers’ appraisals of the voice (Sorrell et al. 2010; Peters et al. 2012; Paulik 2012; van Oosterhout et al. 2013; León-Palacios et al. 2015) and that power differentials between voice and voice hearer play a substantial role in the compliance with command hallucinations (Barrowcliff and Haddock 2006; Reynolds and Scragg 2010). Though gender and perceptions of masculinity/femininity are known factors influencing various aspects of social interaction including power differentials and social appraisals (Eagly 1987; Maccoby, 1990; Rudman and Glick 2008; Ridgeway 2008), little is known about respective gender differences in the voice hearing experience. The only study explicitly investigating gender differences in voice appraisals and interrelating with voices in a quantitative design found more powerful emotional reactions to voices as well as a tendency to respond to them in a more resistant manner in women (Hayward et al. 2016b). Furthermore, the study found that women appraised their voices as being more omnipotent, malevolent, and dominant compared to men (Hayward et al. 2016b). However, the study had a number of methodical limitations, and although gendered relating styles are not stable but depend strongly on the gender category membership of each interaction partner (Jacklin and Maccoby 1978), they did not account for differences due to variations in the interactional constellations, e.g., male voice hearer on male voice vs. male voice hearer on female voice, etc.

The present study aims to:

1. Replicate the aforementioned findings of gender differences in voice appraisals
2. Give a comprehensive account of gender-specific differences in the phenomenology of voices
3. Investigate gender differences in the perception of dangerous command hallucinations
4. Investigate gender differences in appraisals of power differentials and beliefs about voices in different constellations of voice-voice hearer dyads
5. Explore the role of stereotyped masculine and feminine traits in the perception of voices and power differentials between voice and voice hearer both in men and women

## Method

### Participants and procedure

Study participants were recruited from psychiatric in- and outpatient services as well as day care units in and around Vienna (Department of Psychiatry and Psychotherapy of the Medical University of Vienna, Department of Psychiatry and Psychotherapy of the University Hospital Tulln, Social Psychiatric Center of the Caritas Vienna, PSD - Psychosocial Services Vienna). Eligible patients with schizophrenia spectrum disorder were informed about the study by their clinical teams and subsequently referred to the research team, where they were provided with the details and requirements of the study. Out of 148 potential study participants, 28 (F: 12, M: 16) declined to take part in the study. One hundred twenty participants provided informed consent and subsequently took part in a semi-structured interview about the phenomenology of their voices. In addition, they filled in standardized self-rating questionnaires on their beliefs about their most dominant male and female voice and a questionnaire on the power differentials in their respective voice-voice hearer interactions. Furthermore, the voice hearers’ individual masculine/feminine traits were recorded using a standardized self-rating scale. Two participants were unable to identify a dominant voice during the interview, and 1 participant had to quit the study due to a sudden deterioration of their clinical state. These 3 participants were thus excluded from all statistical analyses. The final analyzed sample included a total of 117 participants, 54 of which were female. Participants had a median age of 33 (range: 19–84) and had been hearing voices for a median of 10.5 years (range: 0–45). Diagnoses were obtained from either participants or hospital notes and were as follows: 106 schizophrenia and 11 schizoaffective disorder.

Inclusion criteria included being aged 18 or over, having a diagnosis of a schizophrenia spectrum disorder according to ICD-10, and having experienced verbal acoustic hallucinations within the last month of recruitment in order to control for recall bias. Exclusion criteria were the inability to provide informed consent and a lack of proficiency in German language. The authors assert that all procedures contributing to this work comply with the ethical standards of the relevant national and institutional committees on human experimentation and with the Helsinki Declaration of 1975, as revised in 2008. All procedures involving human subjects/patients were approved by the Ethics Committee of the Medical University of Vienna (Ref: 1342/2013) and the Ethics Committee of Lower Austria (Ref: 316/2015). Written informed consent was obtained from all subjects/patients.

### Measures

#### Demographic and clinical variables

Demographic and clinical variables including age, sex, family status, social network, education, job status, living arrangements, age at manifestation of disease, number of inpatient stays and current medical treatment were assessed using a self-report questionnaire.

#### Symptom severity and clinical impression

The Clinical Global Impression-Schizophrenia Scale (Busner and Targum 2007) (CGI-SCH) was used to assess symptom severity on the dimensions positive symptoms (CGI-pos), negative symptoms (CGI-neg), depressive symptoms (CGI-dep), cognitive symptoms (CGI-cog), and overall severity (CGI-total). The CGI-SCH is a brief and valid clinical assessment, used both in daily clinical practice as well as in clinical research (Haro et al. 2003; Busner and Targum 2007).

#### Phenomenology and characteristics of voices

The auditory hallucination scale of the Psychotic Symptom Rating Scale (PSYRATS-AH) (Haddock et al. 1999) and additional items assessing the number of voices, their gender and age, the voices’ form of address, possible familiarity of the most prominent voices, and the gender of dangerous commanding voices (i.e., voices that commanded voice hearers to harm themselves or others) were used to assess phenomenology and characteristics of VAH. The PSYRATS-AH is a semi-structured interview comprising of 11 items covering the dimensions frequency, duration, location, loudness, beliefs of origin of voices, amount of negative content of voices, amount and intensity of distress, and disruption to life caused by voices as well as controllability of voices.

The auditory hallucination scale of the PSYRATS is a widely used and valid measure with a strong interrater reliability (Drake et al. 2007; Kronmüller et al. 2011) and an adequate test-retest reliability (Drake et al. 2007).

#### Beliefs about voices

The Beliefs about Voices Questionnaire-Revised (BAVQ-R) (Chadwick et al. 2000) was used to assess beliefs about auditory hallucinations as well as participants’ emotional and behavioral responses to them on 5 dimensions, i.e., malevolence, benevolence, omnipotence, resistance, and engagement. In total, the BAVQ-R contains 35 items that are self-rated on a on a 4-point scale ranging from disagree to strongly agree. High internal consistencies of the subscales and adequate construct validity have been reported (Chadwick et al. 2000; Hacker et al. 2008). Participants were asked to rate the BAVQ-R for their most prominent voice (male or female) and for their most prominent voice of the respective other gender. For the present study, the original BAVQ-R was translated to German according to WHO guidelines (Sartorius and Janca 1996) and validated. It showed high internal consistencies for the subscales malevolence (*a* = 0.83), benevolence (*a* = 0.91), resistance (*a* = 0.85), and engagement (*a* = 0.87), but a low internal consistency for the subscale omnipotence (a = 0.62). Test-retest reliability was satisfactory.

#### Power differentials between voice and voice hearer

Power differentials between voice and voice hearer were measured using the Voice Power Differential Scale (Birchwood et al. 2000; Birchwood et al. 2004) (VPD), a brief and reliable self-report measure assessing voice hearers’ perception of disparity of power between themselves and their voices. Voice hearers compare themselves and their voices on six dimensions: strength, confidence, respect, ability to inflict harm, superiority, and knowledge. Participants were asked to rate the VPD for their most prominent voice (male or female) and their most prominent voice of the respective other gender. For the present study, the original VPD was translated according to WHO guidelines (Sartorius and Janca 1996) and validated. It showed favorable psychometric properties with an internal consistency of *a* = 0.833 and a test-retest reliability of *r* = 0.858.

#### Feminine and masculine traits

We measured “feminine” (expressive/communal) and “masculine” (instrumental/agentic) traits using the German Version of the Extended Personal Attribute Questionnaire (Runge et al. 1981) (GEPAQ). The GEPAQ is a self-report scale measuring “masculine” and “feminine” traits on 5 subscales (F+, positive stereotyped female attributes; M+, positive stereotyped male attributes; F-, negative stereotyped female attributes; M-, negative stereotyped male attributes; and M-F, mixed attributes) and is rated on a 6-point scale. The GEPAQ shows good reliability in all subscales except F- (Runge et al. 1981; Athenstaedt et al. 2008). For the present study, only the scales F+ and M+ were used due to findings of a tendency to answer items from the M- scale in accordance with social acceptance and findings of the afore mentioned limited validity of the F- score (Sieverding & Alfermann 1992). The F+ scale (=expressivity scale) contains 8 items rating communal traits that are typically associated with femininity (being kind, being helpful to others, being emotional, being devoted to others, being warm in relation to others, being aware of the feelings of others, being understanding, being gentle). The M+ scale (=instrumentality scale) contains 7 items rating agentic traits that are typically associated with masculinity (being self-confident, feeling superior, making decisions easily, being active, being independent, withstanding pressure, not giving up easily). Hereinafter, the term “instrumentality” or “instrumental traits” will be used for stereotyped masculine agentic traits, and the term “expressivity” or “expressive traits” will be used for stereotyped feminine communal traits.

### Data analysis

Data on overall demographic and clinical characteristics were calculated and presented descriptively using frequency analysis with absolute numbers and percentages. Depending on data distribution, means with standard deviations or medians with range are reported. Comparisons between males and females were calculated using Mann-Whitney *U* tests or chi-square tests, depending on the respective data characteristics. Gender differences of the gender of the dominant voice and gender of dangerous voices were calculated with crosstabs and chi-square tests. To evaluate phenomenological differences between men and women as well as male and female voices and to investigate gender differences in beliefs about voices and power differentials, the ordinal scaled results from the respective measures (PSYRATS, BAVQ-R, VPD) were calculated using Mann-Whitney *U* tests. Correlation analysis was used to evaluate associations between feminine/masculine traits and BAVQ subscores as well as VPD full and item scores. All analyses were performed using two-tailed tests with *α* = 0.05. In order to control for multiple testing, results from hypothesis testing were corrected using Benjamini-Hochberg procedure (Benjamini and Hochberg 1995).

## Results

Male and female voice hearers did not differ in overall sociodemographic and clinical characteristics except for “living arrangements” (*p* = 0.019). No significant gender differences were found for clinical impression and severity of disease as measured by the CGI overall and subscores (Table 1). The investigated sample showed a mean overall CGI score of 3.97 (Table 1), which equals a moderately ill sample according to Haro et al. [25]. CGI overall and subscores did not correlate with numbers of voices heard and were not significantly associated with the gender of the dominant voice or any of the BAVQR or VPD domains. At the time of the investigation, our sample had heard voices for a median of 10.5 years (range: 0–45). Chronicity of voice hearing (i.e. years since onset of voice hearing) did not differ between the genders and was not significantly associated with any BAVQR or VPD domains. Furthermore, no association between voice gender and chronicity could be detected.

**Table 1:** Sociodemographic and basic clinical data of the overall study sample as well as the female and male participants (*N* = 117)

Variable	Overall sample (*n* = 117)		Female participants (*N* = 54)		Male participants (*N* = 63)		*p* value
	Median	Range	Median	Range	Median	Range	
Age in years	33	19–84	35,5	19–84	33	19–59	n.s.
Age in years at illness onset	21	4–48	21	5–48	21	4–45	n.s.
Years of hearing voices	10,5	0–45	11	0–45	10	0–45	n.s.
Number of friends	3	0–300	3	0–20	3	0–300	n.s
Gender	N	%	N	%	N	%	
Female	54	46.2%	–	–	–	–	
Male	63	53.8%	–	–	–	–	
Family status			n.s				
Single	90	76.9%	37	68.5%	53	84.1%	
Married/in partnership	15	12.8%	10	18.5%	5	7.9%	
Divorced or separated	12	10.3%	7	13.0%	5	7.9%	
Social network			n.s				
None or little	27	23.1%	13	24.1%	14	22.2%	
Short-term acquaintance	21	17.9%	9	16.7%	12	19.0%	
Few friends	29	24.8%	11	20.4%	18	28.6%	
Sufficient	40	34.2%	21	38.9%	19	30.2%	
Living arrangements			0.019				
With parents	21	18.1%	7	13%	14	22.6%	
Own household (with partner, etc.)	24	20.7%	18	33.3%	6	9.7%	
Own household alone	56	48.3%	24	44.4%	32	51.6%	
Shared accommodation	9	7.8%	4	7.4%	5	8.1%	
Supervised living	6	5.2%	1	1.9%	5	8.1%	
Working status in current or last job			n.s				
Apprentice	8	7%	1	1.9%	7	11.5%	
Unskilled worker	21	18.3%	9	16.7%	12	19.7%	
Skilled worker	13	11.3%	4	7.4%	9	14.8%	
Employee/public official	39	33.9%	22	40.7%	17	27.8%	
Self-employed	1	0.9%	0	0%	1	1.6%	
Freelance	3	2.6%	1	1.9%	2	3.3%	
Other	30	26.1%	17	31.5%	13	21,3%	
Current working situation			n.s.				
Employed/sick leave	4	3.4%	1	1.9%	3	4.8%	
Unemployed/sick leave	14	12.1%	6	11.1%	8	12.9%	
Retired	49	42.2%	23	42.6%	26	41.9%	
Homemaker	2	1.7%	1	1.9%	1	1,6%	
Student	6	5.2%	5	9.3%	1	1,6%	
Minimum income	16	13.8%	6	11.1%	10	16.1%	
Unemployment benefit	12	10.3%	4	7.4%	8	12.9%	
Other	13	11.2%	8	14.8%	5	8.1%	
Highest education			n.s.				
Special needs school	4	3.4%	1	1.9%	3	4.8%	
Compulsory school	15	12.8%	7	13%	8	12.7%	
Vocational school	39	33.3%	16	29.6%	23	36.5%	
Middle school	34	29.1%	14	25.9%	20	31.7%	
University	24	20.5%	15	27.8%	9	14.3%	
Other	1	0.9%	1	1.9%	0	0%	
Clinical global Impression	Mean	SD	Mean	SD	Mean	SD	
CGI positive symptoms	4.29	0.938	4.42	0.992	4.18	0.885	n.s
CGI negative symptoms	3.70	0.959	3.84	0.969	3.59	0.938	n.s
CGI depressive symptoms	3.42	1.108	3.58	1.230	3.30	0.989	n.s
CGI cognitive symptoms	3.51	1.227	3.56	1.343	3.48	1.134	n.s
CGI overall severity	3.97	0.879	4.12	0.913	3.85	0.833	n.s

### Gender differences in the phenomenology of voices

Men heard significantly more male than female dominant voices (female dominant voice: *n* = 13, 20.6%; male dominant voice: *n* = 47, 74.6%; *p* ≤ 0.001), whereas the gender ratio of dominant voices was balanced in women (female dominant voice: *n* = 23, 42.6%; male dominant voice: *n* = 26, 48.1%; *p* = 0.668). Analyzing the full sample, a significant preponderance of male dominant voices (female dominant voice: *n* = 36, 30.8%; male dominant voice: *n* = 73, 62.4%; *p* ≤ 0.001) was found. Only 8 participants (f: *n* = 5, 9.3%; m: *n* = 3, 4.8%) heard undifferentiated voices that were perceived as neither male nor female.

While men and women did not differ in the intensity of distress or disruption of life caused by their voices, women had a significantly greater amount of distress caused by their voices (F: mean = 2.74, SD = 1.306; M: mean = 2, SD = 1.320; *p* = 0.001), and when voices were heard, they persisted for significantly longer periods of time (F: mean = 2.69, SD = 1.301; M: mean = 2.03, SD = 1.307; *p* = 0.007). Furthermore, women perceived their voices as coming from a place significantly closer to their heads than did men (F: mean = 2.06, SD = 1.309; M: mean = 2.73, SD = 1.461; *p* = 0.011). No gender differences were found for frequency, loudness, controllability, voices’ form of address, attribution of voices to real-life acquaintances, negative voice content, or delusional attribution of voices (Table 2).

**Table 2:** Single items of PSYRATS for the overall population as well as for female and male participants (mean ± SD)

	Overall sample Mean ± SD (*N* = 117)	Female Mean ± SD (*N* = 54)	Male Mean ± SD (*N* = 63)	*p* value
Frequency	2.22 ± 1.293	2.4 ± 1.321	2.08 ± 1.261	0.181
Duration	2.33 ± 1.339	*2.69 ± 1.301*	2.03 ± 1.307	*0.007*
Location	2.42 ± 1.428	2.06 ± 1.309	*2.73 ± 1.461*	*0.011*
Loudness	1.88 ± 0.836	2.02 ± 0.866	1.76 ± 0.797	0.101
Beliefs (origin of voices)	2.44 ± 1.284	2.30 ± 1.298	2.56 ± 1.272	0.299
Amount of negative content	2.50 ± 1.311	2.70 ± 1.312	2.33 ± 1.295	0.087
Degree of negative content	2.07 ± 1.335	2.31 ± 1.322	1.87 ± 1.324	0.083
Amount of distress	2.34 ± 1.359	*2.74 ± 1.306*	2.00 ± 1.320	*0.001*
Intensity of distress	1.98 ± 1.246	2.26 ± 1.277	1.75 ± 1.177	0.019
Disruption to life caused by voices	1.76 ± 1.018	1.96 ± 0.980	1.59 ± 1.026	0.039
Controllability of voices	2.97 ± 1.239	2.96 ± 1.176	2.98 ± 1.299	0.538

### Gender differences in command hallucinations

**Fig. 1:**
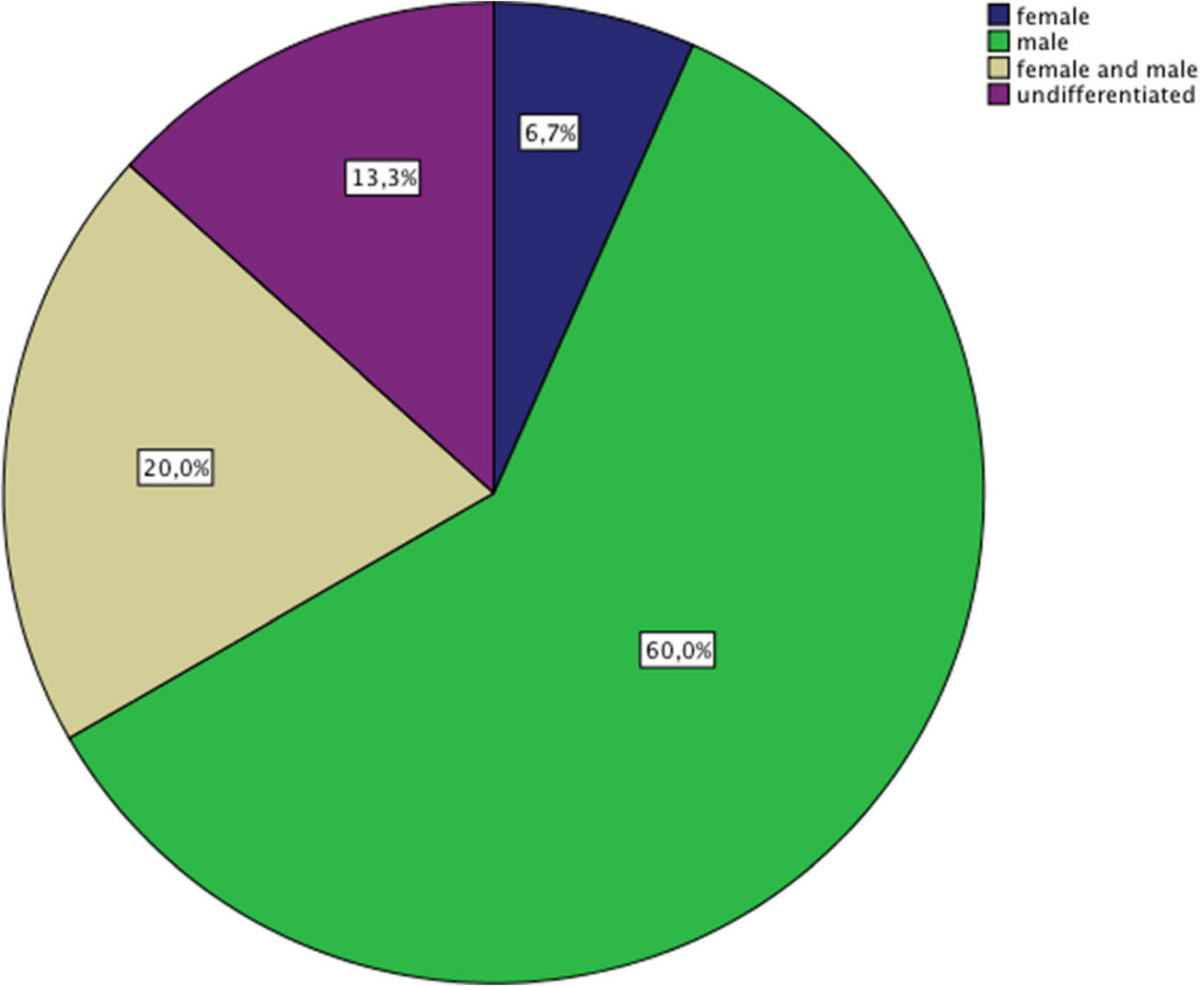
Gender of hallucinations commanding to harm others

We did not detect any gender differences in the frequency of hallucinations that commanded patients to either harm themselves (F: *n* = 26, 48.1%; M: *n* = 28, 44.4%; *p* = 0.748) or others (F: *n* = 13, 24.5%; M: *n* = 18, 27.7%; *p* = 0.587), nor did we find gender differences in the frequency of attempted suicides (F: n = 13, 32.5%; M: *n* = 15, 33.3%; *p* = 0.935) or self-harming behavior (F: *n* = 17, 42.5%; M: *n* = 19, 42.2%; *p* = 0.979). Furthermore, we did not find any significant differences in the gender of voices encouraging self-harm (*p* = 0.684). Voices that encouraged participants to harm others, however, were predominantly male (*n* = 18, 60%; *p* = 0.022) (Fig. 1).

### Beliefs about voices

We did not find any significant gender differences in the appraisals of voices in the domains malevolence (F: mean = 1.552, SD = 0.970; M: mean = 1.514, SD = 1.018; *p* = 0.752), benevolence (F: mean = 1.111, SD = 1.079; M: mean: 1.116, SD = 1.097; *p* = 0.916), and omnipotence (F: mean = 0.819, SD = 0.745; M: mean = 1.618, SD = 0.772; *p* = 0.118) of voices. Furthermore, there were no significant gender differences in the emotional or behavioral responses to voices. When investigating effects of the gender of the dominant voice in relation to the gender of the voice hearer, however, we found that men perceive male dominant voices significantly more malevolent than female dominant voices (female voices: mean = 0.847, SD = 0.842; male voices: mean = 1.681, SD = 0.930; *p* = 0.007). In women, no such effects of voice gender were found.

Significant negative correlations were found between instrumental traits in women and the perception of omnipotence of voices (*r* = −0.368, *p* = 0.007), i.e., high levels of women’s instrumental traits correlated with low levels of perceived voice omnipotence. Effects of instrumentality in men could be shown for male voice hearer on male dominant voice dyads, where we found positive correlations with the perception of benevolence of voices (*r* = 0.382, *p* = 0.009) and emotional engagement with voices (*r* = 0.473, *p* = 0.001), but not for other constellations, i.e., high levels of instrumentality correlated with high levels of perceived benevolence of voices as well as high levels of emotional engagement with voices. Expressive traits did not show any significant correlations with any of the domains of voice appraisal.

### Power differentials between voice and voice hearer

Although we did not detect any differences in the perception of power differentials according to the gender of the voice hearer, all domains of the VPD, except the domain knowledge, were significantly correlated with masculine traits (instrumentality) in the overall sample, i.e., high levels of instrumental traits correlated significantly with high levels of perceived power, strength, self-confidence, respect, superiority, and ability to harm in relation to the voice. Furthermore, there was a significant correlation between instrumentality and VPD overall scores (Table 3). When male and female voice hearers were analyzed separately, a significant correlation between the VPD domain superiority and instrumental traits was detected in men. In females, significant correlations were found for the VPD full score as well as all VPD subdomains except the domain harm, i.e., high levels of instrumental traits in females correlated significantly with high levels of perceived power in relation to the voice (Table 3). Expressivity did not show any significant effects on perceptions of power differentials in the overall sample or in males. In females, however, expressivity showed a significant correlation with low levels of perceived power in relation to the voice (Table 3).

**Table 3:** Significant correlations between masculinity and VPD scores for the dominant voice

GEPAQ_Mpos - Full sample	Pearson’s *r*	*p* value
VPD power dynamic	−0.252	0.007
VPD strength	−0.286	0.002
VPD self-confidence	−0.350	0.000
VPD respect	−0,213	0,025
VPD harm	−0.250	0.009
VPD superiority	−0.330	0.000
VPD total	−0.362	0.000
GEPAQ_Mpos - Males	Pearson’s *r*	*p* value
VPD Superiority	−0.353	0.005
GEPAQ_Mpos - Females	Pearson’s *r*	*p* value
VPD power dynamic	−0.284	0,043
VPD strength	−0.354	0.010
VPD self-confidence	−0.382	0.006
VPD respect	−0.351	0.012
VPD knowledge	−0.340	0.016
VPD superiority	−0.283	0.044
VPD total	−0.466	0.001
GEPAQ_Fpos - Females	Pearson’s *r*	*p* value
VPD power dynamic	0.392	0.004
GEPAQ_Mpos - Participants with male dominant voice	Pearson’s *r*	*p* value
VPD power dynamic	−0.292	0.013
VPD strength	−0.359	0.002
VPD self-confidence	−0.389	0.001
VPD superiority	−0.406	0.000
VPD total	−0.420	0.000
GEPAQ_Mpos - Male participants with male dominant voice	Pearson’s *r*	*p* value
VPD superiority	−0.439	0.002
GEPAQ_Mpos - Female participants with male dominant voice	Pearson’s *r*	*p* value
VPD strength	−0.493	0.012
VPD total	−0.525	0.007

If participants heard a male dominant voice, perceptions of power differentials in the domains power, strength, superiority, self-confidence, and total VPD correlated significantly with participants’ instrumentality scores (Table 3). No effects of instrumentality were found in participants that heard a female dominant voice (Table 3).

When we calculated effects of instrumentality in male on male/male on female and female on male/female on female voice-voice hearer dyads, we found a significant negative correlation between instrumentality and perceptions of superiority in male voice hearers with a male voice (Table 3); i.e., in men, high levels of instrumental traits correlated significantly with high levels of perceived superiority if voices were male, but not if voices were female. Furthermore, we detected significant negative correlations between instrumentality and the VPD domain strength as well as the VPD full score in female on male dyads. No significant correlations were detected for other dyadic constellations (Table 3).

## Discussion

This study aimed to give a comprehensive account on gender differences in the phenomenology and appraisals of VAH and to investigate the impact of gender on power differentials between voice and voice hearer.

In line with a body of evidence suggesting that VAH are typically experienced as a male’s voice (Nayani and David 1996; Legg and Gilbert 2006; McCarthy-Jones et al. 2014), we found a significant preponderance of male dominant voices in our sample. However, when we investigated the gender of the dominant voice in male and female voice hearers separately, we found that, while men did hear significantly more male than female dominant voices, the gender ratio of dominant voices in women was balanced. This is in contrast to a previous study by Nayani and David (1996) that found a preponderance of male dominant voices not only in male but also in female voice hearers. Due to the conflicting evidence, the question whether male and female voice hearers differ in terms of the gender of their dominant voices cannot be conclusively answered and is in need of further replication. Nevertheless, the consideration of our findings in conjunction with etiological models of voice hearing suggesting auditory hallucinations (AH) to be caused by dysfunctional self-monitoring of inner speech (Badcock 2016) poses some interesting questions for respective neurobiological models and is in line with recent findings that a large majority of inner speech (i.e., inner reading voices) resembles the characteristics of the reader’s own speaking voice (Vilhauer 2017). In this context, a tendency towards perceiving verbal hallucinations as congruent with one’s own gender seems plausible and could account for the findings of a preponderance of male dominant voices in males; however, at the same time, it raises some questions concerning the lack of a respective preponderance of female dominant voices in females. In her seminal paper of 2010, Johanna Badcock (2010) put forward a potential neurobiological explanation for the preponderance of male voices in AH that suggested abnormal functioning in the anterior auditory pathway and, more specifically, the right anterior superior temporal gyrus, which is distinctively activated when a female voice is processed in the male brain (Sokhi et al. 2005). Alternative explanations could arise from findings that suggest that female voices have more complex vocal characteristics and require greater integration compared with male voices (Waters and Badcock 2009), leading to a perceptional bias and male misattribution of voices. For example, Chhabra et al. (2012) showed that differences between schizophrenic patients and controls exist in the ability to use timbre-based cues in a voice discrimination task. Since timbre, along with pitch, is a key variable in the discrimination of the gender identity of voices (Ko et al. 2006; Baumann and Belin 2010; Pisanski and Rendall 2011; Latinus and Taylor 2012), such deviations in basic sensory processing could play a role in the attribution of the gender of hallucinated voices (Badcock and Chhabra 2013).

Another fruitful strand of research has focused on the effects of trauma and early sexual abuse on the development and embodiment of AH. As demonstrated by Corstens and Longden (2013), the content of 94% of the voices heard by patients diagnosed with schizophrenia was related to earlier emotionally overwhelming events. In many cases, voices and adverse events shared common emotions such as anger, shame, or guilt as well as common protagonists, e.g., a past abuser. If we consider etiological models of voice hearing proposing that AH result from intrusions from memory in conjunction with the finding that a vast majority of abusers are men (Dubé and Hébert 1988; Cortoni et al. 2017), it seems consistent that their voices may be over-represented in voice hearing.

Although basic phenomenological characteristics of voices such as loudness, frequency, controllability, and amount of negative content were very similar in men and women, women showed greater amounts of distress caused by the voices as well as a persistence of voices for longer periods of time, irrespective of the gender of the voice heard. To date, the body of evidence on gender differences in stress responsivity is conflicting, and both increased and decreased emotional reactivity/stress sensitivity have been reported in women (Riecher-Rössler et al. 2018). As pointed out by Riecher-Rössler (2018), these inconsistencies may arise from methodological differences, type of stress stimulus, and possibly also women’s estradiol level fluctuations during the menstrual cycle, which are known to effect stress response. To our knowledge, our study is the only study testing gender differences with regard to distress and life disruption for AH specifically, and therefore, findings are in need for replication. Confounding factors such as reporting biases (i.e., men’s tendency to underreport symptoms), as depicted for other psychiatric disorders (Sigmon et al. 2005), were not accounted for in our study and should be considered in future studies.

Furthermore, our data suggests that women perceive their voices more frequently from inside and/or closer to their head than men. This contrasts findings of McCarthy-Jones et al. (2015) and also requires further replication. Underlying factors influencing the suggested gender differences in the externalization of voices remain unclear.

We report, for the first time, that one of the most distressing and dangerous subgroups of voices, AH that command the voice hearer to harm others (Shawyer et al. 2003; Birchwood et al. 2014), is predominantly male. This is in line with the findings from a large body of evidence that investigated gender differences in aggressive behavior and suggests stronger tendencies for the externalization of aggression as well as more direct aggressive behaviors in men (Denson et al. 2018; Zaroff and D’Amato, 2015). Considering the role of possible top-down mechanisms and the role of prior expectations in voice identity processing (Clark 2013; Badcock 2016), stereotypes of masculinity (i.e., the aggressive male) may inform voice gender attribution in aggressive hallucinations with a tendency to perceive them as male entities.

Investigating gender differences in voice appraisals in different constellations of voice-voice hearer dyads, we found that male voice hearers experience male voices as significantly more malevolent than female voices, while female voice hearers rated their voices high in malevolence irrespective of their gender (female voices: mean = 1.520, SD = 0.894; male voices: mean = 1.576, SD = 1.095). Numerous studies have pointed towards higher general rates of emotional reactivity as well as higher levels of hostile attributional bias in women (Mathieson et al. 2011), which is in line with the aforementioned finding of women’s high ratings for perceived malevolence independent of voice gender. Though there is some evidence that gender-specific emotional reactivity to social cues is influenced not only by the gender of the perceiver but also the gender of the expresser (Wiggert et al. 2015), respective studies on the specifics of verbal social content (as delivered in VAH) are lacking and pose an interesting field for future studies.

Stereotypical masculine traits (i.e., instrumentality) correlated significantly with various aspects of perceptions of power between voice and voice hearer; i.e., participants with high instrumentality scores perceived themselves as more powerful compared to their most dominant voice. Furthermore, instrumentality correlated positively with perceptions of benevolence as well as emotional engagement in male voice hearers that perceived a male dominant voice. High levels of instrumentality in women were associated with perceiving their dominant voice as less omnipotent, irrespective of the voice’s gender. Stereotypical feminine traits (i.e., expressivity) had limited impact on perceptions of power or voice appraisals and seem to be of negligible relevance for these aspects of voice hearing.

Considering the extensive evidence for positive clinical outcomes associated with voice hearers’ perceived relative power (Birchwood et al. 2000; Barrowcliff and Haddock 2006; Paulik 2012) and the strong association between perceptions of power and instrumentality in our study, we suggest the integration of therapeutic components that strengthen instrumental traits (e.g., assertiveness training or self-esteem work) into overall therapeutic concepts for voice hearing to be feasible targets for therapeutic interventions. In this context, relating therapy, a therapeutic intervention that targets voice hearers’ interpersonal relating and assertiveness strategies, has been tested in a pilot randomized controlled trial and was shown to be effective in reducing auditory hallucination distress (Hayward et al. 2016a). AVATAR therapy, a novel therapeutic method where voice hearers engage in face-to-face dialogue with a digital representation (avatar) of their persecutory voice, also targets the development of instrumental traits in terms of helping voice hearers to reclaim power within the relationship and work on self-esteem and negative self-attributions (Ward et al. 2020). In a typical AVATAR therapy session, the voice hearer is exposed to verbatim critical or hostile hallucinatory content via the digital voice representation (avatar) while being supported by the therapist to respond assertively, e.g., make a self-affirming statement or call the avatar out on exaggerating its power (Ward et al. 2020). A randomized controlled trial investigating the effect of AVATAR therapy on verbal hallucinations compared with a supportive counseling control group showed a reduction in the severity of verbal hallucinations with a large effect size (Craig et al. 2018). To our knowledge, there has been no research on effects of CBT manuals for psychosis on instrumental traits specifically. One study by Hall and Tarrier (2003), however, found that a cognitive behavioral intervention specifically targeted to improve self-esteem, as an adjunct to treatment as usual, resulted in clinical benefits in terms of increased self-esteem, decreased psychotic symptomatology, and improved social functioning.

One limitation of the present study was the relatively low statistical power to detect gender differences with small or even medium effect sizes in comparisons that involved specific subgroups of our sample, e.g., the gender-wise evaluation of different dyadic voice-voice hearer constellations. This may have led to type 2 error, especially in calculations in the “male voice hearer on female dominant voice” subgroup, where case numbers were particularly low.

Furthermore, due to the nature of the sample, our findings cannot be generalized to non-schizophrenic groups of voice hearers.

## Conclusion

In summary, the current study contributes to a deeper understanding of gender differences and the role of “masculine” and “feminine” traits in the voice hearing experience. It is the first study to investigate different constellations of gender membership within interactional voice-voice hearer dyads and suggest respective effects of specific gendered voice-voice hearer constellations.

Our findings have a number of interesting implications for etiological models of voice hearing as well as clinical implications. It should be highlighted that, even though we found similar basic phenomenological voice characteristics in both genders, women experienced significantly more subjective distress caused by the voices. This adds an additional risk factor for unfavorable clinical outcomes in women. Furthermore, we found that instrumentality correlated significantly with the perception of power differentials between voice and voice hearer; i.e., high levels of instrumental traits correlated with high levels of perceived power, strength, self-confidence, etc. in relation to the voice. This effect was particularly pronounced in women. Considering the extensive evidence for positive clinical outcomes associated with voice hearers’ perceived relative power (Birchwood et al. 2000; Barrowcliff and Haddock 2006; Paulik 2012), we suggest the integration of therapeutic components that strengthen instrumental traits (e.g., assertiveness training or self-esteem work) into overall therapeutic concepts and psychotherapeutic/psychological interventions for voice hearing. This should be considered especially in the treatment of women.

## Data Availability

The data that support the findings of this study are available from the corresponding author (S.SK) upon reasonable request.
